# Mavacamten Cardiac Myosin Inhibitor: Clinical Applications and Future Perspectives

**DOI:** 10.7759/cureus.82722

**Published:** 2025-04-21

**Authors:** Mahmoud M Ramadan, Raghad A Al-Najjar, Rokia S Abady, Hala A Obaid, Yasmeen A Mostafa, Mohammad T Al-Obeid, Mohammed Elmahal

**Affiliations:** 1 Cardiology, Faculty of Medicine, Mansoura University, Mansoura, EGY; 2 Clinical Sciences, College of Medicine, University of Sharjah, Sharjah, ARE; 3 Medicine and Surgery, Public Health Centre, Dubai Health Authority, Sharjah, ARE; 4 Diabetes and Endocrinology, Khartoum North Teaching Hospital, Khartoum, SDN

**Keywords:** diastolic, heart failure, hypertrophic cardiomyopathy, mavacamten, myosin inhibitor, preserved ejection fraction

## Abstract

Mavacamten is a first-in-class, selective allosteric inhibitor of cardiac myosin adenosine triphosphatase (ATPase) that has emerged as a novel therapeutic option for patients with symptomatic hypertrophic obstructive cardiomyopathy (HOCM) who remain refractory to conventional therapy, such as beta-blockers and calcium channel blockers. Clinical trials have demonstrated that mavacamten reduces left ventricular outflow tract (LVOT) obstruction, improves diastolic function, and enhances exercise capacity, thereby addressing the key pathophysiological mechanisms underlying HOCM. However, its use requires careful dose titration due to the potential for reversible reductions in left ventricular ejection fraction (LVEF). While mavacamten represents a meaningful therapeutic advancement in HOCM management, its role in other conditions such as non-obstructive hypertrophic cardiomyopathy (HCM) and heart failure with preserved ejection fraction (HFpEF) remains investigational. Preliminary data suggest favorable effects on diastolic parameters and cardiac biomarkers in HFpEF, but larger studies are needed to establish efficacy. While these challenges persist, ongoing research is exploring broader cardiomyopathy populations, optimization of combination therapies, and development of novel formulations. As a targeted modulator of myocardial contractility, mavacamten exemplifies the shift toward mechanism-based, precision therapeutics in cardiovascular medicine.

## Introduction and background

Mavacamten is a first-in-class, selective allosteric inhibitor of cardiac myosin adenosine triphosphatase (ATPase), developed primarily for the treatment of hypertrophic obstructive cardiomyopathy (HOCM) [1,2]. HOCM is characterized by asymmetric left ventricular (LV) hypertrophy, typically involving the interventricular septum, that leads to dynamic left ventricular outflow tract (LVOT) obstruction. This obstruction impairs systolic ejection and contributes to symptoms such as exertional dyspnea, chest pain, palpitations, and reduced exercise capacity. By modulating the hypercontractile sarcomeric state underlying this obstruction, mavacamten has been shown to improve functional capacity, reduce LVOT gradients, and enhance quality of life in patients with symptomatic HOCM [3,4].

In addition to obstructive disease, hypertrophic cardiomyopathy (HCM) encompasses non-obstructive phenotypes, which represent a significant subset of cases. These are similarly characterized by myocyte disarray, diastolic dysfunction, and hyperdynamic contractility in the absence of LVOT gradients. Owing to these shared pathophysiological features, mavacamten is being investigated in other myocardial conditions marked by impaired relaxation and sarcomeric hyperactivity. This rationale has extended interest to heart failure with preserved ejection fraction (HFpEF), a heterogeneous syndrome associated with increased myocardial stiffness, impaired diastolic filling, and elevated sarcomeric tension. Although current data are preliminary, they suggest potential benefits of mavacamten in reducing filling pressures and improving cardiac biomarker profiles in HFpEF.

Ongoing research is also exploring mavacamten in combination with established heart failure therapies, such as β-blockers, renin-angiotensin-aldosterone system (RAAS) inhibitors, and anti-arrhythmic agents. These strategies may augment efficacy or minimize adverse effects in selected subpopulations. Nonetheless, several uncertainties persist, including the long-term effects on myocardial energetics, fibrosis progression, and arrhythmic risk.

This narrative review provides a comprehensive appraisal of the pharmacologic mechanisms, current clinical applications, and future therapeutic directions of mavacamten.

## Review

Search strategy and sources

This narrative review is based on a comprehensive literature search conducted across PubMed, Scopus, and ClinicalTrials.gov databases, covering the period from January 2018 to March 2025. Search terms included combinations of keywords such as “mavacamten”, “cardiac myosin inhibitor”, “hypertrophic cardiomyopathy”, “HOCM”, “HFpEF”, “myosin ATPase inhibition”, and “sarcomere-targeted therapy”. Preference was given to peer-reviewed original articles, phase II/III clinical trials, review articles, and mechanistic preclinical studies. Additionally, conference abstracts and registry updates from major cardiovascular meetings (e.g., American College of Cardiology (ACC), European Society of Cardiology (ESC), and American Heart Association (AHA)) were included to ensure incorporation of the most recent findings, particularly those from ongoing or recently completed studies such as ODYSSEY-HCM, EMAGINE-HFpEF, and MAVA-LTE. Reference lists of key studies were also screened for relevant publications. The search was limited to English-language sources.

Historical background

The pharmacological management of cardiomyopathy has undergone significant evolution, shaped by a deeper understanding of myocardial contractility and energy dynamics. The development of mavacamten reflects a shift away from non-selective inotropic stimulation toward mechanism-specific modulation of sarcomeric activity. Its emergence signifies a targeted, disease-modifying strategy in the management of HCM, particularly in patients with obstructive physiology who are refractory to conventional therapies [1]. Beyond its ventricular effects, mavacamten may also influence atrial remodeling indirectly by reducing LVOT gradients and improving ventricular compliance. Although no direct antifibrotic action has been confirmed, its potential to alleviate chronic atrial stretch remains an area of active investigation [5].

Concurrently, increasing awareness of social determinants of health has brought attention to disparities in diagnosis, treatment access, and outcomes in conditions such as HCM and HFpEF. Socioeconomic status, healthcare access, and comorbid conditions substantially impact the real-world use and benefit of novel therapies like mavacamten. Incorporating equity-focused frameworks into drug development and implementation strategies is therefore essential [6].

Chemical structure, pharmacokinetics, and mechanism of action

Mavacamten is a first-in-class, small-molecule, allosteric inhibitor of cardiac myosin [7]. Structurally, it contains a diphenyl ether core substituted at the para position [1,2]. It exhibits high plasma protein binding (>97%) and favorable oral bioavailability (>95%), with low systemic clearance and a prolonged elimination half-life of six to nine days - properties that support once-daily oral dosing [8].

It is primarily metabolized by cytochrome P450 2C19, with minor contributions from CYP3A4 and CYP2C9 [8]. Genetic polymorphisms in CYP2C19 can lead to substantial interindividual variability in plasma drug concentrations, necessitating individualized dose titration and therapeutic monitoring. Elimination is primarily fecal (76%), with renal clearance accounting for approximately 24% [8]. Table 1 summarizes these pharmacokinetic properties.

**Table 1:** Pharmacokinetic parameters of mavacamten in humans.

Parameter	Value	Notes
Oral bioavailability	>95%	Supports once-daily oral dosing; minimal first-pass metabolism
Plasma protein binding	98–99%	High binding; potential for displacement interactions with other highly bound drugs
Volume of distribution	Extensive	Distributes well into tissues, indicating broad tissue penetration
Primary metabolism	CYP2C19 (major); CYP3A4, CYP2C9 (minor)	Dose adjustment is required in poor or ultra-rapid metabolizers
Elimination half-life	6–9 days	Varies with CYP2C19 metabolic phenotype, allows for once-daily dosing; necessitates careful titration to steady-state
Clearance	Low	Facilitates prolonged systemic exposure, may contribute to drug accumulation if not monitored
Route of elimination	Fecal (~76%), renal (~24%)	No need for renal dose adjustment in most cases
Genetic variability impact	High	Pharmacogenomic variability affects metabolism; therapeutic drug monitoring is recommended

Mechanistically, mavacamten inhibits cardiac myosin ATPase, reducing the number of force-generating actin-myosin cross-bridges during systole. This leads to decreased myocardial contractility and alleviates LVOT obstruction in HOCM by reducing systolic anterior motion (SAM) of the mitral valve [8].

A key preclinical finding is mavacamten’s stabilization of the “super relaxed state” (SRX) of cardiac myosin, a conformation that sequesters myosin heads along the thick filament, rendering them inactive [9]. This reduces adenosine triphosphate (ATP) consumption, improves myocardial energetic efficiency, enhances diastolic relaxation, and reduces oxygen demand, particularly in hypertrophied myocardium [10].

Effects on cardiac muscle contraction

Cardiac contraction depends on calcium dynamics, sarcomere length, and adrenergic stimulation. In healthy myocardium, β1-adrenergic stimulation enhances contractility through calcium mobilization, but this also increases oxygen demand and arrhythmogenic risk [11-13].

Mavacamten reduces hypercontractility through a distinct, non-adrenergic, calcium-independent mechanism. By stabilizing the SRX state, it decreases the number of active myosin heads, reducing sarcomere tension without increasing intracellular calcium or sympathetic tone [14]. This mechanism contrasts with traditional inotropes and avoids their associated risks [15,16].

These features make mavacamten particularly suited for HOCM, where systolic function is preserved but diastolic filling is impaired due to sarcomeric hypercontractility [17,18]. The drug improves diastolic function and LVOT hemodynamics without compromising overall systolic performance.

Therapeutic implications and clinical applications

Mavacamten is approved by the U.S. FDA for adults with symptomatic obstructive HCM (New York Heart Association (NYHA) class II-III) who remain symptomatic despite optimal medical therapy, such as β-blockers or non-dihydropyridine calcium channel blockers [19]. Approval was based on pivotal phase 3 trials showing significant improvements in functional status and LVOT gradient reduction. Table 2 summarizes key outcomes.

**Table 2:** Summary of key clinical trial outcomes supporting mavacamten use in HOCM. * All p < 0.001 vs. placebo; ^† ^8.9% vs. 17.9% with placebo. HOCM: hypertrophic obstructive cardiomyopathy; OLE: open-label extension; RCT: randomized controlled trial; SRT: septal reduction therapy; VO₂: oxygen consumption.

Trial (phase)	Design	Population	Primary outcome	Result	Reference
EXPLORER-HCM (3)	RCT, 30 weeks	n = 251, HOCM	∆ peak VO₂	+1.4 mL/kg/min*	[20]
VALOR-HCM (3)	RCT, 56 weeks	SRT-eligible (n = 112)	SRT avoidance	50% reduction†	[21]
MAVA-LTE (OLE)	Open-label extension	n = 231	Safety/sustainability	Sustained efficacy at 104 weeks	[22]

These trials demonstrated that mavacamten improves exercise capacity, reduces symptom burden, and can delay or prevent the need for septal reduction therapy in appropriately selected patients. However, functional improvements were generally modest compared to surgical myectomy. Subgroup analyses suggest patients with less severe gradients or high ejection fractions may derive limited benefit, underscoring the importance of careful patient selection.

Mavacamten's negative inotropic effects require cautious titration and ongoing echocardiographic monitoring. As part of the FDA-mandated Risk Evaluation and Mitigation Strategies (REMS) program, therapy must be paused or adjusted if left ventricular ejection fraction (LVEF) falls below 50%, as transient reductions have been observed in a small subset of patients during trials [23,24]. Although currently approved only for symptomatic obstructive HCM, mavacamten is under investigation for broader indications, including non-obstructive HCM (MAVERICK-HCM) [5] and HFpEF (EMBARK-HFpEF) [25], where its sarcomere-targeted mechanism may offer therapeutic benefit.

Dose titration and LVEF monitoring

Due to mavacamten’s negative inotropic properties, appropriate dose titration and LVEF monitoring are essential to ensure safety and treatment efficacy. The drug demonstrates a steep concentration-response relationship, with higher plasma levels potentially resulting in transient reductions in LVEF. To address this, the FDA has implemented a REMS program [26], requiring (a) baseline and serial echocardiograms to assess LVEF at regular intervals, (b) initiation at a fixed starting dose, followed by individualized titration based on echocardiographic and clinical response, and (c) temporary treatment interruption or dose reduction if LVEF falls below 50%.

These safeguards are based on trial observations, where a minority of patients experienced asymptomatic or symptomatic reductions in LVEF that were reversible upon dose adjustment or drug discontinuation. This structured approach supports safe use of mavacamten in real-world clinical settings while mitigating the risk of systolic dysfunction [21,23,27].

Hypertrophic cardiomyopathy

HCM is now clinically categorized into obstructive (HOCM) and non-obstructive subtypes. HOCM, marked by dynamic LVOT obstruction, affects approximately 60% of symptomatic patients [21]. Mavacamten is currently approved for use in adults with symptomatic HOCM (NYHA class II-III) who remain symptomatic despite optimal guideline-directed medical therapy [23,28].

Evidence for mavacamten’s efficacy stems from multiple clinical trials that collectively demonstrated improvements in functional capacity, LVOT gradients, and quality of life. Table 3 summarizes the key design features and outcomes from pivotal studies supporting its clinical use.

**Table 3:** Summary of major clinical trials evaluating mavacamten in HCM. HCM: hypertrophic cardiomyopathy; HOCM: hypertrophic obstructive cardiomyopathy; SRT: septal reduction therapy; VO₂: oxygen consumption; NYHA: New York Heart Association; LVOT: left ventricular outflow tract; KCCQ: Kansas City Cardiomyopathy Questionnaire; NT-proBNP: N-terminal pro-B-type natriuretic peptide; LVEF: left ventricular ejection fraction.

Trial	Design	Population	Primary endpoint	Key results
MAVERICK-HCM [5]	Phase 2, double-blind	59 symptomatic non-obstructive HCM patients	Safety, biomarker changes	Well tolerated; reductions in NT-proBNP and troponin I observed
MAVA-LTE [22]	Long-term extension of EXPLORER	EXPLORER and PIONEER enrollers	Durability of effect, safety	Sustained improvements in LVOT gradients and NYHA class; stable LVEF with long-term use
EXPLORER-HCM [23]	Phase 3, randomized, double-blind	251 symptomatic HOCM patients (NYHA II–III)	Composite of peak VO₂ + NYHA class improvement	37% met primary endpoint vs. 17% placebo; 74% had ≥50 mmHg gradient reduction
PIONEER-HOCM [24]	Phase 2, open-label dose-ranging	21 symptomatic HOCM patients	Safety, LVOT gradient reduction	Demonstrated early safety and LVOT gradient reduction; informed individualized titration strategy
VALOR-HCM [28]	Phase 3, randomized, placebo-controlled	Patients referred for septal reduction therapy (SRT)	Proportion undergoing or remaining eligible for SRT	17.9% proceeded to SRT vs. 76.8% placebo; reduced need for invasive therapy
ODYSSEY-HCM [29]	Phase 3, ongoing	Symptomatic non-obstructive HCM (n = 150 planned)	Change in KCCQ score, VO₂, NT-proBNP	Ongoing; designed to evaluate symptomatic and functional benefit

These trials collectively support mavacamten’s role in reducing LVOT obstruction, improving functional capacity (e.g., NYHA class and peak oxygen consumption (VO₂)), and lowering the need for invasive procedures in obstructive HCM. The long-term extension study (MAVA-LTE) confirmed that benefits are durable with continued treatment, and no new safety signals have emerged [30].

In contrast, mavacamten’s role in non-obstructive HCM is still investigational. MAVERICK-HCM showed favorable safety and biomarker trends [25], while ODYSSEY-HCM is evaluating symptom and exercise improvements in this population [29]. Although mavacamten is effective following β-blocker or calcium channel blocker failure, it is not currently approved for combination use with these agents, and no synergistic effects have been confirmed [27]. Future studies are needed to explore combination strategies, real-world use across broader populations, and its role in comorbid conditions such as atrial fibrillation or hypertension.

Heart failure with preserved ejection fraction

HFpEF remains a major therapeutic challenge, accounting for approximately 40-50% of all heart failure cases [31]. It is defined by the presence of classic heart failure symptoms and signs, a preserved LVEF ≥50%, and echocardiographic or biomarker evidence of diastolic dysfunction or structural cardiac abnormalities. Key pathophysiological traits include ventricular stiffness, impaired myocardial relaxation, microvascular inflammation, myocardial fibrosis, and elevated left atrial pressure, particularly during exertion. These features contribute to exertional dyspnea, reduced exercise tolerance, and frequent hospitalizations, particularly among elderly patients with multiple comorbidities [32,33].

Traditional therapeutic strategies that target neurohormonal pathways or hemodynamic loading conditions, such as mineralocorticoid receptor antagonists (TOPCAT) [32] or angiotensin receptor-neprilysin inhibitors (PARAGON-HF) [33], have shown limited or modest benefit in HFpEF, likely due to the syndrome's heterogeneity. This highlights the need for mechanism-specific therapies that address underlying myocardial abnormalities. Mavacamten, as a selective cardiac myosin ATPase inhibitor, may offer a novel therapeutic approach by reducing sarcomeric tension, improving myocardial compliance, and lowering diastolic wall stress. By directly targeting the hypercontractile sarcomere, mavacamten may improve ventricular filling dynamics and alleviate symptoms in a mechanistically defined HFpEF subgroup.

The EMBARK-HFpEF trial, a phase 2, single-arm, open-label study, evaluated mavacamten in 30 patients with HFpEF and LVEF ≥60% [25]. Over 26 weeks, patients demonstrated a 26% reduction in N-terminal pro-B-type natriuretic peptide (NT-proBNP), a 13% reduction in high-sensitivity troponin T, and a 20% reduction in troponin I, suggesting attenuation of myocardial wall stress and subclinical injury. Additionally, 41.7% of participants improved by at least one NYHA class, and echocardiographic indices of diastolic function (e.g., E/e′ ratio and left atrial volume) showed modest improvement. However, mean LVEF declined by 3.2%, which, while within an acceptable safety margin, reinforces the need for close systolic function monitoring, especially in patients with limited contractile reserve.

Despite these promising physiologic and symptomatic signals, several limitations temper the generalizability of the EMBARK trial findings [25], including a small sample size (n = 30), short study duration (26 weeks), open-label design, and absence of a placebo control group. These factors limit causal inference and underscore the need for larger, randomized, placebo-controlled trials with event-driven endpoints to determine the long-term efficacy and safety of mavacamten in HFpEF.

In comparison to established therapies, such as angiotensin receptor-neprilysin inhibitors (ARNIs) (e.g., sacubitril/valsartan), which reduce NT-proBNP and atrial pressure [33], and sodium-glucose cotransporter 2 (SGLT2) inhibitors (e.g., dapagliflozin, empagliflozin), which have demonstrated benefits in hospitalization reduction and symptom control [34], mavacamten offers a sarcomere-targeted alternative with a potentially unique mechanism of benefit (Table 4). Whether it yields additive or synergistic effects in combination therapy is unknown and warrants future investigation.

**Table 4:** Comparison of mavacamten with existing HFpEF therapies. HFpEF: heart failure with preserved ejection fraction; ARNIs: angiotensin receptor-neprilysin inhibitors; SGLT2: sodium-glucose cotransporter-2.

Drug	Mechanism	Diastolic effect	Clinical evidence
Mavacamten	Sarcomere hypercontractility	Direct (myosin inhibition)	Phase 2 only [25]
ARNIs	Neurohormonal blockade	Minimal	Negative (PARAGON-HF) [33]
SGLT2 inhibitors	Metabolic modulation	Indirect (fibrosis reduction)	Phase 3 (EMPEROR-Preserved) [34]

No studies to date have evaluated the impact of mavacamten on arrhythmia burden in HFpEF. While early safety data do not indicate pro-arrhythmic effects, the absence of rhythm monitoring in the EMBARK trial precludes definitive conclusions [25]. Further studies with continuous electrocardiographic monitoring are necessary to address this knowledge gap.

Efficacy and safety outcomes

The clinical efficacy and safety of mavacamten have been evaluated across several rigorously conducted trials in patients with symptomatic HOCM, including VALOR-HCM [28], EXPLORER-HCM [35], and the long-term MAVA-LTE extension study [30]. Collectively, these trials demonstrated clinically meaningful improvements in exercise capacity, symptom burden, and hemodynamic parameters, with an acceptable safety profile.

In the EXPLORER-HCM trial [23], which enrolled adults with NYHA class II-III symptoms, mavacamten significantly improved peak VO₂ by 1.4 ± 0.3 mL/kg/min compared to 0.0 ± 0.3 mL/kg/min in the placebo group (P < 0.0001). The Kansas City Cardiomyopathy Questionnaire (KCCQ) shortness-of-breath scores improved by 15 ± 8 points versus 5 ± 6 points with placebo (P < 0.001), and 60% of treated patients experienced at least one NYHA functional class improvement. Additionally, 74% of patients receiving mavacamten achieved a ≥50 mmHg reduction in LVOT gradient compared to 28% in the placebo group.

The VALOR-HCM trial [28], which focused on patients referred for septal reduction therapy (SRT), found that mavacamten reduced the proportion of patients proceeding to SRT at 32 weeks (8.9% vs. 17.9%), highlighting its potential to delay or obviate the need for invasive intervention in appropriately selected individuals. Long-term efficacy and safety were confirmed in the MAVA-LTE extension study [22], which demonstrated sustained improvements in symptoms, LVOT gradients, and quality-of-life indices over 104 weeks. Importantly, there was no evidence of progressive systolic dysfunction or emergent adverse safety signals.

Safety outcomes across these trials have been generally favorable. The most notable adverse event was a reversible reduction in LVEF, observed in a small proportion of patients. These events were managed with dose adjustment or temporary discontinuation, underscoring the importance of routine echocardiographic monitoring and adherence to the FDA-mandated REMS program [26]. A summary of the major efficacy and safety outcomes across these pivotal trials is provided in Table 5.

**Table 5:** Summary of key efficacy and safety outcomes across major mavacamten trials in HOCM. HOCM: hypertrophic obstructive cardiomyopathy; SRT: septal reduction therapy; VO₂: oxygen consumption; NYHA: New York Heart Association; LVOT: left ventricular outflow tract; KCCQ-OSS: Kansas City Cardiomyopathy Questionnaire Overall Summary Score; LVEF: left ventricular ejection fraction.

Trial	Primary endpoint	Key efficacy results	Safety findings
MAVA-LTE [22]	Long-term durability	Sustained improvement in symptoms, LVOT gradient, and functional class at 104 weeks	No new safety concerns identified
EXPLORER-HCM [23]	Change in peak VO₂	• +1.4 mL/kg/min vs. placebo (P < 0.001). • KCCQ-OSS ↑15 points (vs. 5 in placebo). • 60% improved by ≥1 NYHA class	• LVEF <50% in 6% of patients • No proarrhythmia reported
VALOR-HCM [28]	SRT avoidance	• 8.9% vs. 17.9% proceeded to SRT (50% relative reduction). • LVOT gradient ↓ ~45 mmHg	• Transient LVEF reduction in a minority; all reversible

While these findings are promising, it is important to acknowledge that most enrolled patients had moderate symptom burden (NYHA II-III) rather than advanced heart failure, and effect sizes, while statistically and clinically significant, vary by subgroup. Therefore, although mavacamten is a meaningful therapeutic advance for HOCM, ongoing monitoring and patient selection remain critical.

Future perspectives and potential developments

Mavacamten is a first-in-class, selective cardiac myosin inhibitor with emerging potential in cardiovascular therapeutics beyond HOCM. As understanding of its mechanism deepens, future applications may include non-obstructive HCM, HFpEF, and other myocardial conditions characterized by hypercontractility or diastolic dysfunction. There is also growing interest in evaluating mavacamten in combination with established heart failure therapies, such as ARNIs, SGLT2 inhibitors, and beta-blockers. These agents offer different, potentially complementary mechanisms, such as promoting natriuresis, improving vascular compliance, or reducing neurohormonal activation, which could synergize with mavacamten’s sarcomere-directed unloading effects. However, such combination strategies are theoretical at this stage and require formal evaluation in clinical trials.

Several studies are underway or planned to explore the broader applicability of mavacamten. In addition to the completed EMBARK-HFpEF trial [25], ongoing investigations include the ODYSSEY-HCM phase III trial [29] and real-world observational efforts such as the MAVA-Registry, which aim to assess safety, effectiveness, and patient-centered outcomes in more diverse populations, including those with multiple comorbidities or older age.

Current clinical practice guidelines, including the 2023 ESC consensus, assign mavacamten a class IIa recommendation for adults with symptomatic obstructive HCM not adequately managed by beta-blockers or calcium channel blockers [36]. Despite FDA approval, adoption in routine practice is influenced by several factors: the requirement for REMS program enrolment, the need for serial echocardiographic monitoring to assess LVEF, and the cost of therapy [26]. Access and reimbursement remain limited in some regions, contributing to slower uptake outside of specialized centers [37].

Although mavacamten is a small-molecule agent, ongoing innovation may focus on developing extended-release formulations or alternative delivery platforms to improve convenience and adherence. However, such developments remain exploratory. Ultimately, the broader integration of mavacamten into cardiology practice will depend on forthcoming phase III trial results, updated guideline endorsements, real-world cost-effectiveness data, and streamlined implementation protocols to support safety monitoring and access.

Emerging indications

Mavacamten, a selective cardiac myosin ATPase inhibitor, is often referred to as a “myosin inhibitor” due to its unique mechanism of reducing hypercontractility by stabilizing myosin heads in the SRX [9,17]. While its current approval is limited to symptomatic HOCM, several ongoing studies are exploring its utility in broader cardiovascular contexts. One major initiative is the MAVA-Registry (NCT05174403), a prospective, multicenter, open-label registry enrolling approximately 2000 patients with HCM, designed to assess the long-term safety and effectiveness of mavacamten in real-world settings. This registry includes patients with diverse comorbidities and disease phenotypes, providing valuable insight into variability in treatment response, safety profiles in elderly cohorts, and longitudinal outcomes in both obstructive and non-obstructive forms of hypertrophy [30].

Mavacamten is also under investigation in patients with severe symptomatic aortic stenosis (AS), particularly in those with concentric LV hypertrophy and preserved ejection fraction (EF). A pilot study (NCT05722552) is evaluating whether mavacamten can reduce hyperdynamic contraction and improve diastolic filling in AS patients prior to valve replacement [38]. The hypothesized mechanism is that by reducing sarcomeric tension, mavacamten may alleviate diastolic dysfunction and myocardial oxygen demand in patients with pressure overload-induced hypertrophy, like its action in HOCM. Furthermore, trials such as EMBARK-HFpEF (NCT04766892) [25] and ODYSSEY-HCM (NCT05174416) [29] are expanding the scope of investigation to non-obstructive HCM and HFpEF, respectively. In HFpEF, which is often marked by increased myocardial stiffness and impaired ventricular compliance, mavacamten may offer benefit by modulating sarcomere function and improving diastolic filling, as shown in preliminary biomarker and echocardiographic data from EMBARK [25].

On a molecular level, mavacamten’s action is informed by extensive preclinical work on cardiac myosin-binding protein C (cMyBP-C), a key regulator of sarcomere dynamics. Mutations in the MYBPC3 gene are among the most common in familial HCM, and patients with such genotypes may represent a subgroup especially responsive to sarcomere-modifying therapies [10,17]. As such, future personalization strategies may involve genotype-guided treatment selection, sarcomere imaging biomarkers (e.g., echocardiographic strain and cardiac MRI T1 mapping), or pharmacogenomic profiling to optimize therapeutic response. However, as mavacamten expands into new indications, appropriate patient selection remains critical. Caution is warranted in populations with borderline or reduced LVEF, as mavacamten’s negative inotropic properties could exacerbate systolic dysfunction. All ongoing studies employ stringent echocardiographic surveillance and LVEF thresholds to mitigate this risk.

Novel formulations and delivery systems

Although mavacamten is currently formulated as an oral once-daily small molecule with high oral bioavailability (>95%) [8], its pharmacokinetics are influenced by metabolism via CYP2C19, which can vary significantly between individuals. This interpatient variability necessitates individualized dose titration and frequent echocardiographic monitoring to ensure safety, particularly in avoiding excessive negative inotropy and reduction in LVEF. These limitations have led to interest in exploring novel drug delivery platforms that could improve pharmacodynamic consistency, extend duration of action, or reduce the burden of therapeutic monitoring.

Potential future strategies under theoretical or preclinical exploration include (a) extended-release oral formulations, which may help maintain steady plasma levels and reduce peak-trough variability; (b) nanoparticle-encapsulated systems, designed for controlled release and potentially tissue-targeted delivery; (c) depot (injectable) formulations, which could improve adherence in patients requiring long-term therapy by decreasing the frequency of dosing and visits for monitoring; and (d) lipid-based carriers or prodrug approaches, to modify distribution, uptake, or metabolic stability.

While these approaches remain experimental and no human trials have reported results to date, the overarching goal is to develop formulations that maintain therapeutic plasma concentrations with reduced variability and minimized need for intensive monitoring. Such innovations may be particularly relevant for expanding mavacamten’s use beyond specialist centers or in populations where access to serial imaging is limited.

Importantly, the idea that targeted delivery might "treat symptoms without invasive surgery" should be interpreted conservatively. While a well-designed formulation could reduce the need for septal reduction therapy in HOCM by consistently controlling LVOT gradients, this depends on maintaining safe and effective drug exposure, not on bypassing surgery per se. Future delivery innovations should focus on increasing treatment accessibility, optimizing safety margins, and enabling broader, community-level adoption of mavacamten therapy.

## Conclusions

Mavacamten is a significant advance in treating symptomatic HOCM, offering a mechanism-based approach by selectively inhibiting cardiac myosin ATPase to reduce LVOT obstruction, improve diastolic filling, and enhance exercise capacity. Trials like EXPLORER-HCM and VALOR-HCM have shown symptom relief, improved functional class, and reduced need for septal reduction, with durable effects in long-term studies. However, its use requires careful monitoring due to potential LVEF reductions, as outlined in the FDA’s REMS program, and its high cost may limit access outside specialized centers.

While early data suggest promise in non-obstructive HCM and HFpEF, these remain investigational, with current HFpEF evidence limited to small cohorts. Careful patient selection is crucial, especially in those with borderline systolic function or comorbidities. Future studies may refine patient stratification using phenotypic or genotypic predictors.
